# PCAIs stimulate MAPK, PI3K/AKT pathways and ROS-Mediated apoptosis in aromatase inhibitor-resistant breast cancer cells while disrupting actin filaments and focal adhesion

**DOI:** 10.18632/oncotarget.28759

**Published:** 2025-07-29

**Authors:** Jassy Mary S. Lazarte, Kweku Ofosu-Asante, Syreeta L. Tilghman, Nazarius S. Lamango

**Affiliations:** ^1^Florida A&M University College of Pharmacy Pharmaceutical Sciences, Institute of Public Health, Tallahassee, FL 32307, USA

**Keywords:** PCAIs, ROS, MAPK, PI3K/AKT, LTLT-Ca cells

## Abstract

The estrogen receptor is overexpressed in and promotes 67-80% and 90% of female and male breast cancer cases, respectively. Hormone independence, enhanced motility, and signaling by growth factors have been attributed to aromatase inhibitor (AI) resistance and MAPK pathway activation. We used long-term letrozole-treated (LTLT-Ca) breast cancer cells to evaluate polyisoprenylated cysteinyl amide inhibitors (PCAIs) as potential therapies for AI-resistant breast cancer. PCAIs specifically disrupt G-proteins such as KRAS, an upstream regulator of MAPK and PI3K/AKT pathways. PCAIs were tested against the viability, phosphorylation of MAPK and PI3K/AKT pathways, apoptosis, and migration of LTLT-Ca cells. NSL-YHJ-2-27 was potent against LTLT-Ca viability with an EC50 of 4.8 μM. MEK (p-MEK1/2), ERK (p-ERK1/2), and p90RSK (p-p90RSK) phosphorylation were significantly increased by 2-, 2-, and 6.4-fold, respectively. PCAIs increased AKT phosphorylation 36-fold. NSL-YHJ-2-27 at 2, 3 and 5 μM stimulated ROS generation by 4-, 8- and 10-fold, respectively. PCAIs inhibited cell proliferation and colony formation by 95% and 74%, respectively, increased active caspase 7 and BAX 1.5-fold and 56%, respectively. NSL-YHJ-2-27 (10 μM) induced LTLT-Ca spheroid degeneration by 61%. LTLT-Ca cell migration was inhibited by 31 and 80% following treatment with 2 and 5 μM NSL-YHJ-2-27, respectively. NSL-YHJ-2-27 disrupted F-actin filaments, vinculin punctates and levels by 33%. These results indicate that the PCAIs’ activation of the MAPK and PI3K/AKT pathways causes apoptosis, possibly through proapoptotic p-p90RSK isoforms, AKT-induced ROS production or anoikis through disruption of focal adhesion. These effects against LTLT-Ca cells suggest potential PCAIs therapeutic applications against antihormonal-resistant breast cancers.

## INTRODUCTION

Steroid hormones such as androgen, estrogen, and progesterone are directly involved in breast carcinogenesis [1, 2], with most human breast cancers developing as estrogen-dependent and overexpressing estrogen receptors [3]. Breast cancer is the second leading cause of cancer-related mortality in women in the United States. According to the American Cancer Society Cancer Facts and Figures 2025, there will be an estimated 316,950 new invasive breast cancer cases in women and a predicted female fatality of 42,170. In recent years, the success of endocrine therapies such as the aromatase inhibitors (AI) and selective estrogen receptor modulators (SERMs), has lowered the mortality rate of estrogen receptor-positive (ER^+^) breast cancer [4].

Letrozole is a third-generation AI that is typically recommended as first-line endocrine therapy for postmenopausal women diagnosed with ER^+^ breast cancer [5]. While it effectively reduces tumor burden, some patients develop AI resistance, that can progress to metastatic disease and death [6, 7]. AI resistance usually involves increased signaling of human epidermal growth factor receptor type 2 (HER2) and epidermal growth factor receptor (EGFR) [8, 9]. It is commonly associated with epithelial to mesenchymal transition (EMT), which leads to enhanced proliferation, motility, invasiveness [10] and evasion of apoptosis [11] which constitutes a significant therapeutic obstacle. Consequently, identifying novel therapies to overcome resistance is essential for reducing tumor burden. A previous study demonstrated that when MCF-7 cells stably transfected with the human aromatase gene are exposed to letrozole, they adapt to estrogen deprivation by activating alternate kinase signaling pathways resulting in the maintenance of transcription and cell proliferation [8]. To date, several studies have focused on targeted approaches to treat AI-resistant breast cancer [12–16]. The discovery of CDK4/6 inhibitors has improved the treatment options for ER^+^ breast cancer [17]. Due to innate resistance to endocrine therapy (ET) in approximately 20% of patients, combination therapies including CDK4/6 inhibitors and ET have been efficacious in delaying cancer progression and improving survival. These have become the standard of care for advanced ER^+^/HER2- breast cancer [18]. The commercially available and FDA-approved CDK4/6 inhibitors Palbociclib, Ribociclib, and Abemaciclib have different inhibition profiles against CDK isoforms resulting in diverse phenotypic effects on ER^+^ breast cancer [19–21]. However, patients with metastatic disease that rely on monotherapy eventually progress on combination therapy due to intrinsic or acquired resistance to ET, the CDK4/6 inhibitors or to both therapies [22]. Ultimately, new therapies are still sought to meet the needs of patients who develop resistance to current therapies.

Mutated RAS genes have long been associated with tumorigenesis [23]. Among the three isoforms of RAS (HRAS, NRAS, KRAS), aberrant KRAS signaling drives 30% of all human cancers and 5% of breast cancer [24]. Significant evidence has shown that oncogenic RAS proteins drive hyperactivation of upstream RAS activators such as receptor tyrosine kinases (RTKs) and perturbations in the activity of RAS regulators such as guanine nucleotide exchange factors (GEFs), and GTPase-activating proteins (GAPs) promote and enhance tumorigenicity [25, 26]. Besides being a potent mediator of tumor progression, hyperactive RAS is also found to confer resistance to breast cancer therapies [27–30]. Therefore, RAS involvement in both tumor progression and resistance provides a valid rationale for the development of RAS-targeted therapies for breast cancer.

Due to almost four decades of futile efforts to develop anti-KRAS agents, the KRAS oncogene had long been regarded as “undruggable” [31]. One principal reason for the challenges faced in this effort is the lack of suitable binding pockets on KRAS to facilitate the binding of small molecule drugs [32]. An alternative approach to addressing the KRAS problem involves the development of small molecular entities that would competitively bind to KRAS binding sites and curtail the intermolecular interactions that are crucial to it’s signaling. The polyisoprenylated cysteinyl amide inhibitors (PCAIs) are designed to specifically target the polyisoprenylation-dependent interactions to suppress the hyperactivities of KRAS and similar G-proteins [33]. Studies in our lab have shown that these compounds activate the phosphorylation of MAPK pathway kinases [34], suppress migration and invasion [35–38], and induce apoptosis [36, 39] in various cancer cell lines including the triple negative breast cancer cell lines, MDA-MB-231 and MDA-MB-468 [40]. While MAPK pathway activation leading to P90RSK phosphorylation activation may lead to apoptosis depending on the P90RSK isoform, activation of the other KRAS signaling arm, PI3K/AKT pathway, though generally prosurvival/oncogenic, has been demonstrated to cause apoptosis upon activation through the generation of reactive oxygen species (ROS) that cause widespread damage to nucleic acids, proteins and lipids [41, 42]. The ability of PCAIs to mitigate various cancer hallmarks in the various cancer cell lines has been well established [33–35, 37–40, 43]. Thus, we aimed to determine whether these agents possess the same therapeutic potential against AI-resistant breast cancer by investigating the impact of PCAIs on cell viability, proliferation, colony formation, migration, and invasion as well as determining the molecular changes involved.

## RESULTS

### NSL-YHJ-2-27 suppresses the viability of LTLT-Ca breast cancer cells

**Figure 1:**
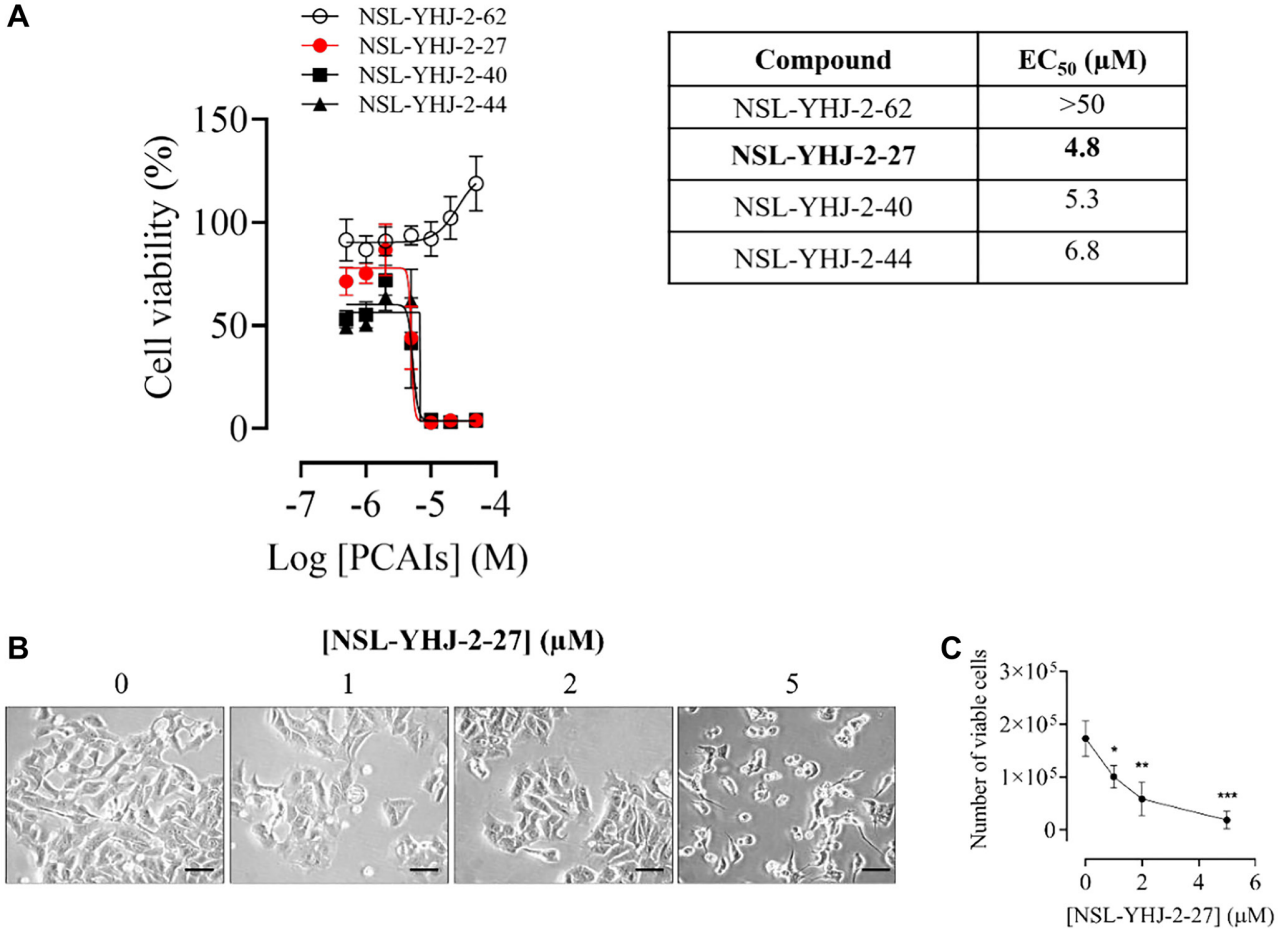
PCAIs inhibit the viability and proliferation of AI-resistant LTLT-Ca breast cancer cells. (**A**) LTLT-Ca cells were treated with the indicated concentrations of respective agents for 48 h. The resazurin reduction assay was performed to determine cell viabilities as described in the methods section. The EC50 values were obtained from nonlinear regression plots. (**B**) Cells were grown in 6-well plates and treated with the indicated concentrations of PCAIs. After treatment, images of the cells were captured using the Keyence microscope at 20× magnification (Scale bar = 100 μm). (**C**) The cells were then trypsinized and viable cells were counted using Countess II automated cell counter. Data are representative of three independent experiments. Statistical significance (^*^ *p* < 0.05; ^**^ *p* < 0.01; ^***^ *p* < 0.001) was determined by one-way ANOVA with post hoc Dunnett’s test.

The PCAIs were designed to target and disrupt the polyisoprenylated-dependent modifications of G-proteins such as KRAS. Since oncogenic KRAS promotes cell survival and proliferation, the effect of the PCAIs on cancer cell viability was determined. The potency of the PCAIs depends on these three key features: 1) the polyisoprenyl moiety, 2) the α-amino N-substituent size, and 3) the N-cycloalkyl ring size. NSL-YHJ-2-62, which does not have the polyisoprenyl moiety, had no effect on the treated cells, displaying an EC_50_ value greater than 50 μM. NSL-YHJ-2-27, NSL-YHJ-2-40, and NSL-YHJ-2-44 show prominent effects against LTLT-Ca cell viability. These PCAIs have three key features but differ in the sizes of α-amino N-substituent sizes and N-cycloalkyl ring. Both NSL-YHJ-2-27 and NSL-YHJ-2-44 have the same α-amino N-substituent size, but the latter has a cycloheptyl ring while the former has a cyclooctyl ring. Furthermore, both NSL-YHJ-2-27 and NSL-YHJ-2-40 have the same cyclooctyl ring, but NSL-YHJ-2-40 has a longer N-substituent size with a 4-carbon chain while NSL-YHJ-2-27 has a 1-carbon chain. The EC_50_ values obtained for NSL-YHJ-2-27, NSL-YHJ-2-40, and NSL-YHJ-2-44 are 4.8, 5.3, and 6.8 μM, respectively, making NSL-YHJ-2-27 the most potent of the four analogs (Figure 1A).

Furthermore, since cell shape and motility are mediated by CDC42 and RAC1 whose interactions are also dependent on polyisoprenylation, the effects of the PCAIs on cell shape, cell migration and invasion were determined. We found that increasing concentrations of PCAIs caused physical changes to the cells. Cells treated with the vehicle, acetone, did not show any changes in appearance. However, those treated with PCAIs shrank with increasing concentrations of PCAIs. LTLT-Ca cells treated with 5 μM showed the most shrinkage and rounding. Half of the cells were dead, visibly floating in the media (Figure 1B). This result indicates that the PCAIs have an effect on the LTLT-Ca cells that causes them to change their physical appearance before death. A key feature of cancer cells is attributed to their ability to proliferate rapidly and form colonies that eventually become tumors. It was observed that the PCAIs caused a significant decrease in the number of viable cells by 46, 66, and 75% when treated with 1, 2, and 5 μM of NSL-YHJ-2-27, respectively (Figure 1C). To conduct further mechanistic studies of the effects of the PCAIs on the LTLT-Ca cells, NSL-YHJ-2-27 was chosen due to its higher potency and lower log *P*-value.

### NSL-YHJ-2-27 activates the phosphorylation of MAPK and PI3K/AKT pathway enzymes in LTLT-Ca cells

**Figure 2:**
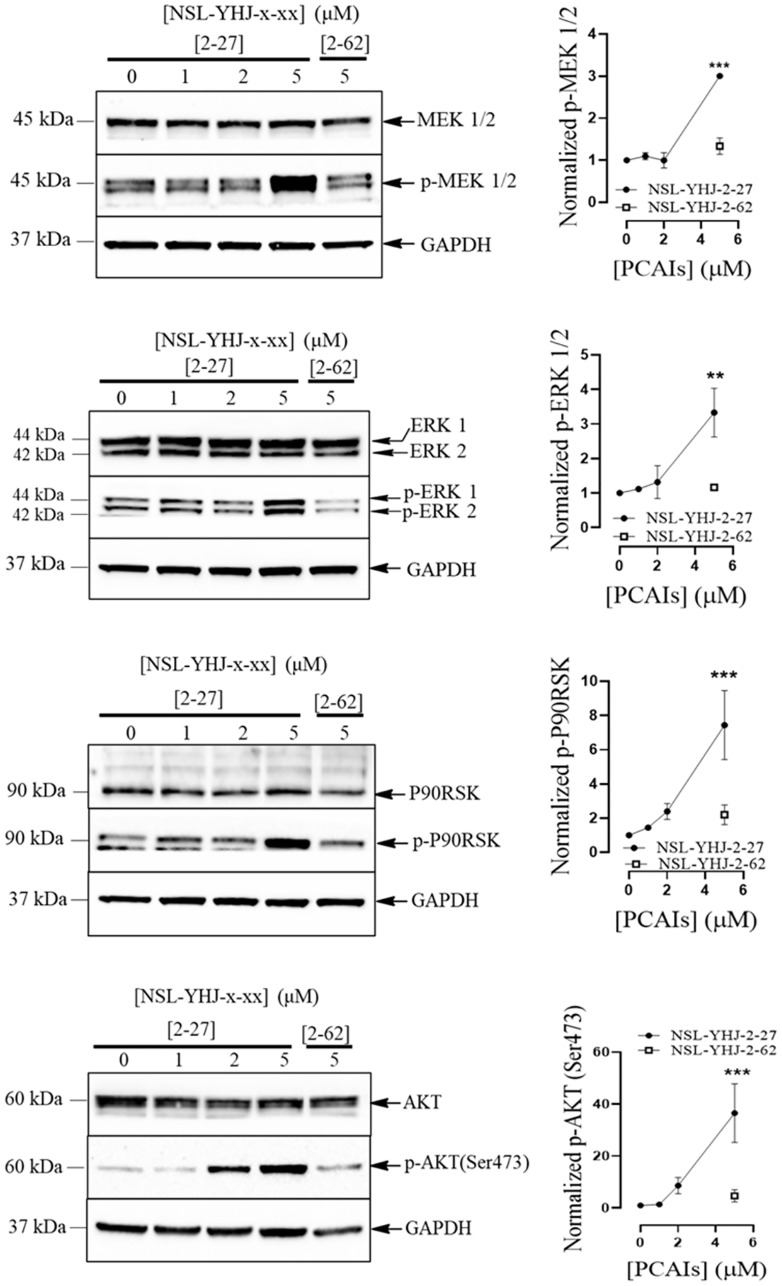
NSL-YHJ-2-27 stimulates the phosphorylation of MAPK and AKT pathway enzymes in AI-resistant cells. LTLT-Ca cells were treated with the indicated concentrations of NSL-YHJ-2-27 or the non-farnesylated analog, NSL-YHJ-2-62 for 48 h. They were then lysed and analyzed by western blotting as described in the methods section. Data are representative of three independent experiments. Statistical significance (^*^ *p* < 0.05; ^***^ *p* < 0.001; ^****^ *p* < 0.0001) was determined by one-way ANOVA with post hoc Dunnett’s test.

The MAPK and PI3K/AKT pathways are major pathways involved in cell proliferation and survival that are often dysregulated in cancers. Here, we evaluated the effects of PCAIs on the phosphorylation of three MAPK pathway kinase enzymes, MEK 1/2, ERK 1/2 and p90RSK and on AKT of the PI3K/AKT pathway. Exposure of LTLT-Ca cells to 5 μM PCAIs remarkably increased the phosphorylation levels of the kinases, MEK 1/2, ERK 1/2 and p90RSK by 2-fold, 2-fold, and 6.4-fold, respectively, relative to the controls. Phosphorylation at Ser 473 of the AKT kinase was significantly increased by 36-fold (Figure 2).

### PCAIs induce the production of reactive oxygen species in LTLT-Ca cells

**Figure 3:**
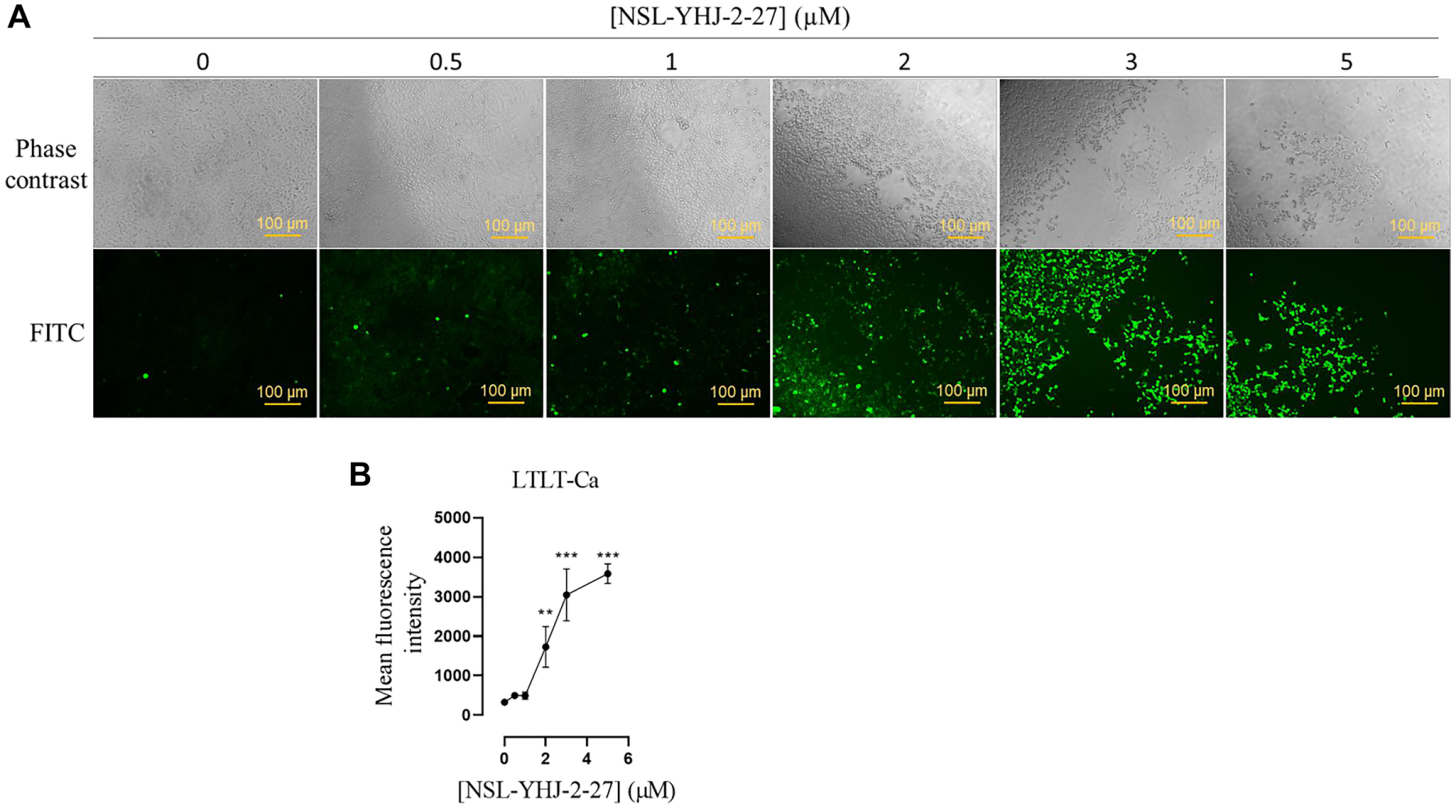
NSL-YHJ-2-27 stimulates the generation of ROS in LTLT-Ca. (**A**) LTLT-Ca cells were treated with 0, 0.5, 1, 2, 3 and 5 μM of NSL-YHJ-2-27 for 24 h. Cells were washed with 1 X PBS and DCFH-DA working solution was introduced and incubated for 45 mins. Fluorescent images of the ROS production in LTLT-Ca were captured with the Keyence BZ-X800 series microscope at 10X magnification. (**B**) Quantification of mean fluorescent intensities was done with Keyence BZ-800 analyzer. The mean fluorescence intensities against varying concentration was plotted using GraphPad Prism. One-Way ANOVA was used to determine statistical significance between increasing concentration of NSL-YHJ-2-27 and mean fluorescence intensities (^**^ *p* < 0.01, ^***^ *p* < 0.001). Data is representative of triplicated experiments.

Studies have shown that AKT hyperphosphorylation results in reactive oxygen species (ROS)-induced apoptosis [42, 44]. ROS such as superoxide and hydroxyl radicals in cells readily react with and damage DNA, lipids, and proteins consequently inhibiting cell growth and/or causing cell death [42, 43]. Here, we show that NSL-YHJ-2-27 treatment induces ROS in LTLT-Ca cells (Figure 3A) as the fluorescence intensity increased 4-, 8- and 10-fold at 2, 3 and 5 μM NSL-YHJ-2-27, respectively (Figure 3B).

### PCAIs attenuate the expression of human epidermal growth factor receptor type 2 and estrogen receptor in LTLT-Ca breast cancer cells

**Figure 4:**
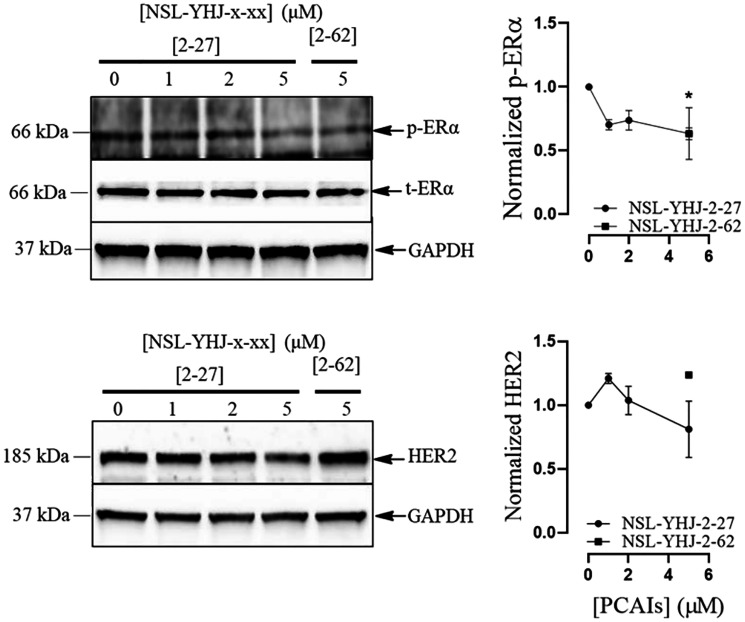
PCAIs effects on HER2 and ERα levels. LTLT-Ca cells were treated with (0-5 μM) concentrations of NSL-YHJ-2-27 or (5 μM) of the non-farnesylated analog, NSL-YHJ-2-62 for 48 h. They were then lysed and analyzed for p-ERα and HER2 levels by western blotting as described in the methods section. Data are representative of three independent experiments. Statistical significance (^*^ *p* < 0.05) was determined by one-way ANOVA with post hoc Dunnett’s test.

The human epidermal growth factor receptor type 2 (HER2) and the estrogen receptor alpha (ERα) are two of the three biomarkers whose overexpression drive receptor-positive breast cancers. We determined the effect of the PCAIs on HER2 and ER levels in the LTLT-Ca cells. After treatment with 5 μM NSL-YHJ-2-27, ERα phosphorylation significantly decreased by 37%. NSL-YHJ-2-27 at 5 μM, caused a marginal but insignificant decrease of HER2 levels of 20% (Figure 4).

### NSL-YHJ-2-27 depletes RAC1 and CDC42 levels

**Figure 5:**
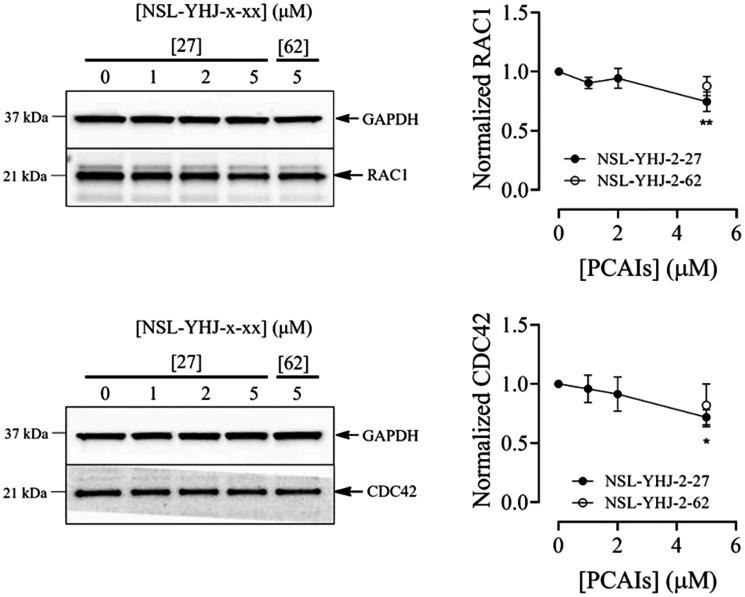
NSL-YHJ-2-27 depletes RAC1 and CDC42 levels in LTLT-Ca cells. LTLT-Ca cells were treated with the indicated concentrations of NSL-YHJ-2-27 or the non-farnesylated analog, NSL-YHJ-2-62 for 48 h. They were then lysed and analyzed by western blotting as described in the methods section. Data are representative of three independent experiments. Statistical significance (^*^ *p* < 0.05; ^**^ *p* < 0.01) was determined by one-way ANOVA with post hoc Dunnett’s test.

Previous reports demonstrated that NSL-YHJ-2-27 depletes the cellular levels of some G-proteins, such as RAC1 and CDC42, in both lung and breast cancer cell lines [37, 43]. In agreement with these previous results, a marginal but significant decrease in RAC1 and CDC42 levels of 25% and 28%, respectively, was also observed in LTLT-Ca cells treated with 5 μM of PCAIs relative to the untreated cells (Figure 5).

### NSL-YHJ-2-27 induces cell death in LTLT-Ca 3D spheroids

**Figure 6:**
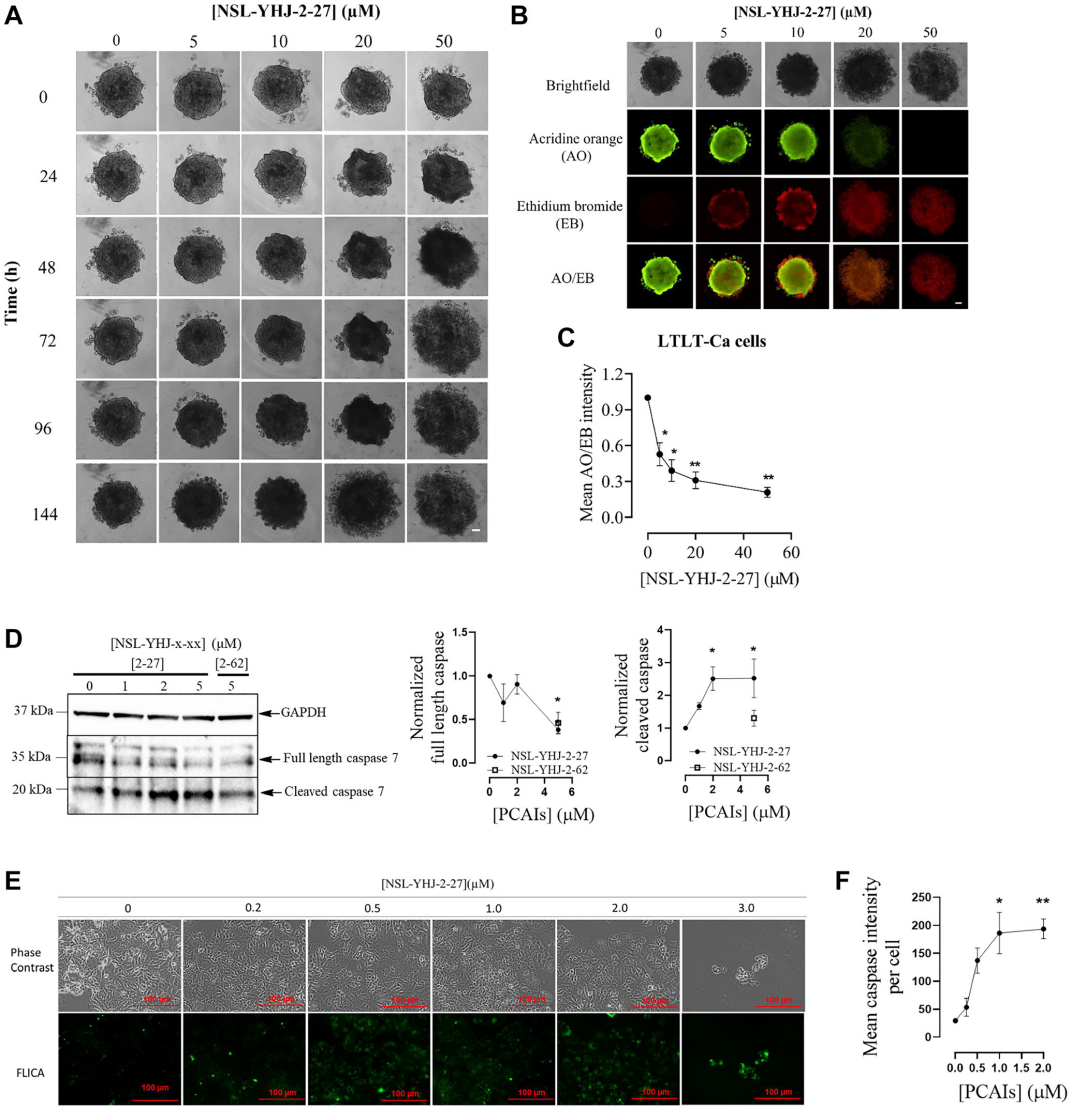
NSL-YHJ-2-27 causes the disintegration of compact 3D LTLT-Ca spheroids and caspase activation in cells. LTLT-Ca cells were seeded in 96U round bottom Nunclon Sphera plate and left to grow for 48 h to form compact spheroids. The compact spheroids were then treated with the indicated concentrations of NSL-YHJ-2-27 and analyzed for 144 h. Spheroids were cultured and treated in triplicates. (**A**) Brightfield images of the spheroids were captured at different time points. (**B**) The spheroids were stained with 10 μg/mL of AO/EB dye mixture at 144 h post-PCAIs treatment and images captured at 4× magnification using Nikon Ti Eclipse microscope (scale bars = 100 μm). Note that the brightfield images taken at 144 h are aligned with the AO/EB-stained images for easier visualization only. (**C**) The fluorescence intensity of both dyes was determined using NIS Element software. The mean AO/EB intensity ratios (equivalent to the viable/dead cells) were calculated, representative of three different independent experiments. (**D**) Western blotting for caspase 7 was performed after lysing LTLT-Ca cells and full length and cleaved caspase levels determined. Three independent experiments were conducted. (**E**). Cells were stained on 8-well μ slide plates (ibidi) after NSL-YHJ-2-27 (0–5 μM) treatments in triplicates using CaspaTagTM caspase-3/7 kit. The fluorescence intensities were measured with the Keyence BZ-X800 fluorescence microscope, analyzed using Keyence BZ-X800 analyzer software. (**F**) Mean fluorescence intensities per cell against respective concentration of NSL-YHJ-2-27 were plotted using Graph Pad Prism. Statistical significance (^*^ *p* < 0.05; ^**^ *p* < 0.01) was determined by one-way ANOVA with post hoc Dunnett’s test.

To evaluate the effect of the PCAIs in 3D cultured LTLT-Ca spheroids, the cells were grown into compact spheroids and treated with varying concentrations of the PCAIs. The results reveal a concentration-dependent cell death in the spheroids. Deterioration of the spheroid was initially observed in cells treated with 50 μM after 24 h with the spheroid totally disintegrating into apparently larger spheres within 72 h (Figure 6A). This was also observed for the 20 μM treatment after 144 h. At 144 h following PCAIs treatment, the spheroids were stained with acridine orange AO/EB to determine the degree of cell death. The mean AO/EB intensity ratios show a concentration-dependent decrease from 42% through 61% and 69% to 79% in cells treated with 5, 10, 20, and 50 μM, respectively (Figure 6B, 6C). In addition, the effect of PCAIs on caspase activity was determined using western blotting. We observed a significant decrease in full length caspase 7 of 95%, alongside a significant 150% increase of active cleaved caspase 7 levels (Figure 6D). The ability of PCAIs to induce apoptosis is further supported by the results obtained using the CaspaTag^™^ Caspase-3,7 *in situ* Assay Kit and fluorescence microscopy to evaluate labelled caspase levels. As the concentration of PCAIs increased, so did the fluorescence intensities; NSL-YHJ-2-27 (2 μM) dramatically raised the amounts of active caspase 5-fold (Figure 6E, 6F). At 3 μM most of the cells detached from the plate resulting in much fewer imaged cells.

### PCAIs induce changes in levels of apoptotic markers in LTLT-Ca cells

**Figure 7:**
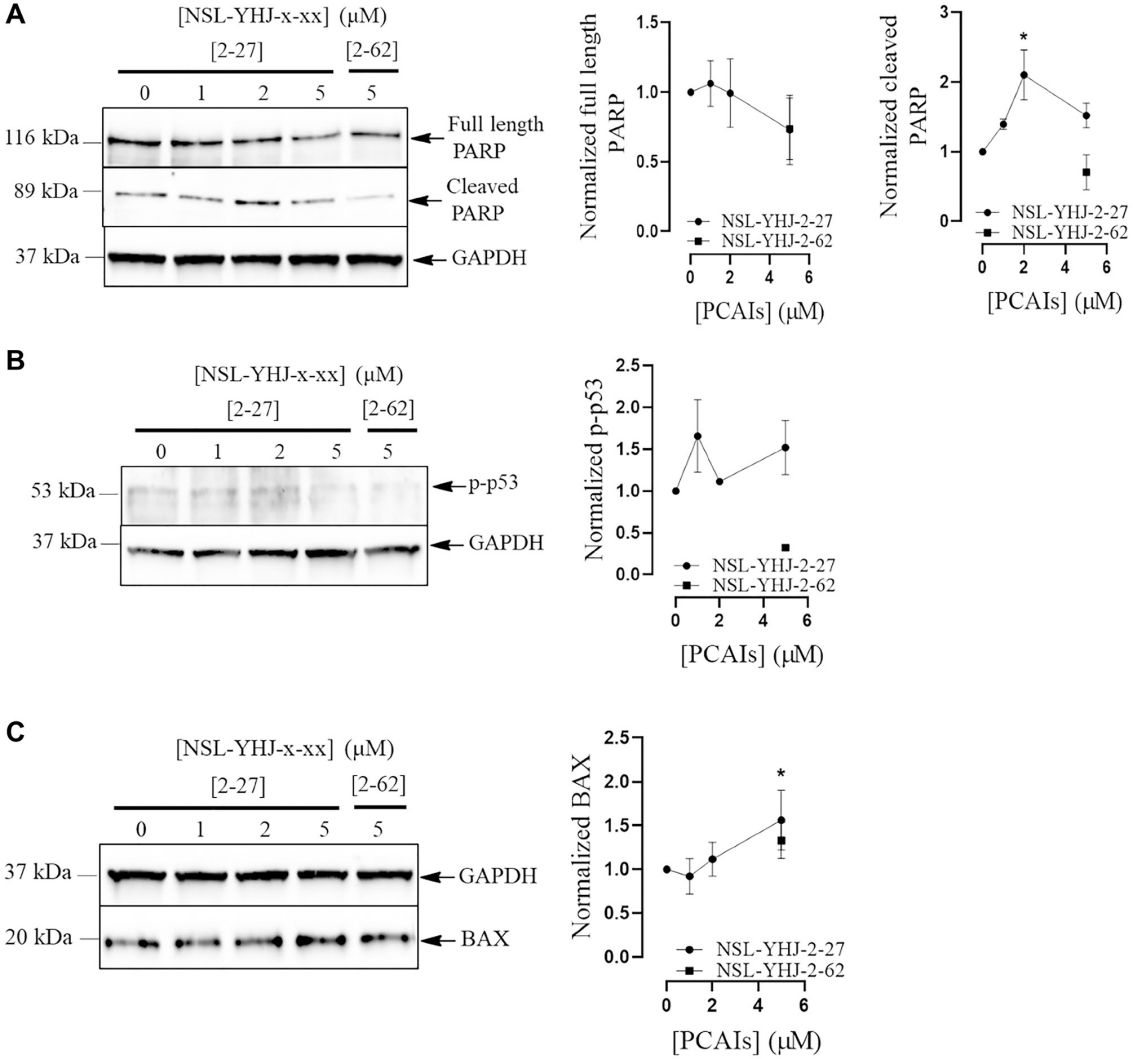
NSL-YHJ-2-27 alter the levels of apoptotic markers. LTLT-Ca cells were treated with the indicated concentrations of NSL-YHJ-2-27 or the non-farnesylated analog, NSL-YHJ-2-62 for 48 h. They were then lysed and analyzed by western blotting for full length and cleaved PARP (**A**), phosphorylated p53 (**B**), and BAX protein levels (**C**) as described in the methods section. Data are representative of three independent experiments. Statistical significance (^*^ *p* < 0.05; ^**^ *p* < 0.01) was determined by one-way ANOVA with post hoc Dunnett’s test.

Apoptotic markers are often investigated by cancer researchers to determine the mechanisms of cancer cell death. BH3-only proapoptotic proteins and tumor suppressors play vital roles in the development of novel cancer therapies. When we evaluated NSL-YHJ-2-27 on LTLT-Ca cells, we noticed a slight 27% decrease in full length PARP (Figure 7A). No significant change in phopho-p53 levels was observed (Figure 7B). However, NSL-YHJ-2-27 significantly increased BAX levels by 56% (Figure 7C).

### NSL-YHJ-2-27 inhibits LTLT-Ca colony formation

**Figure 8:**
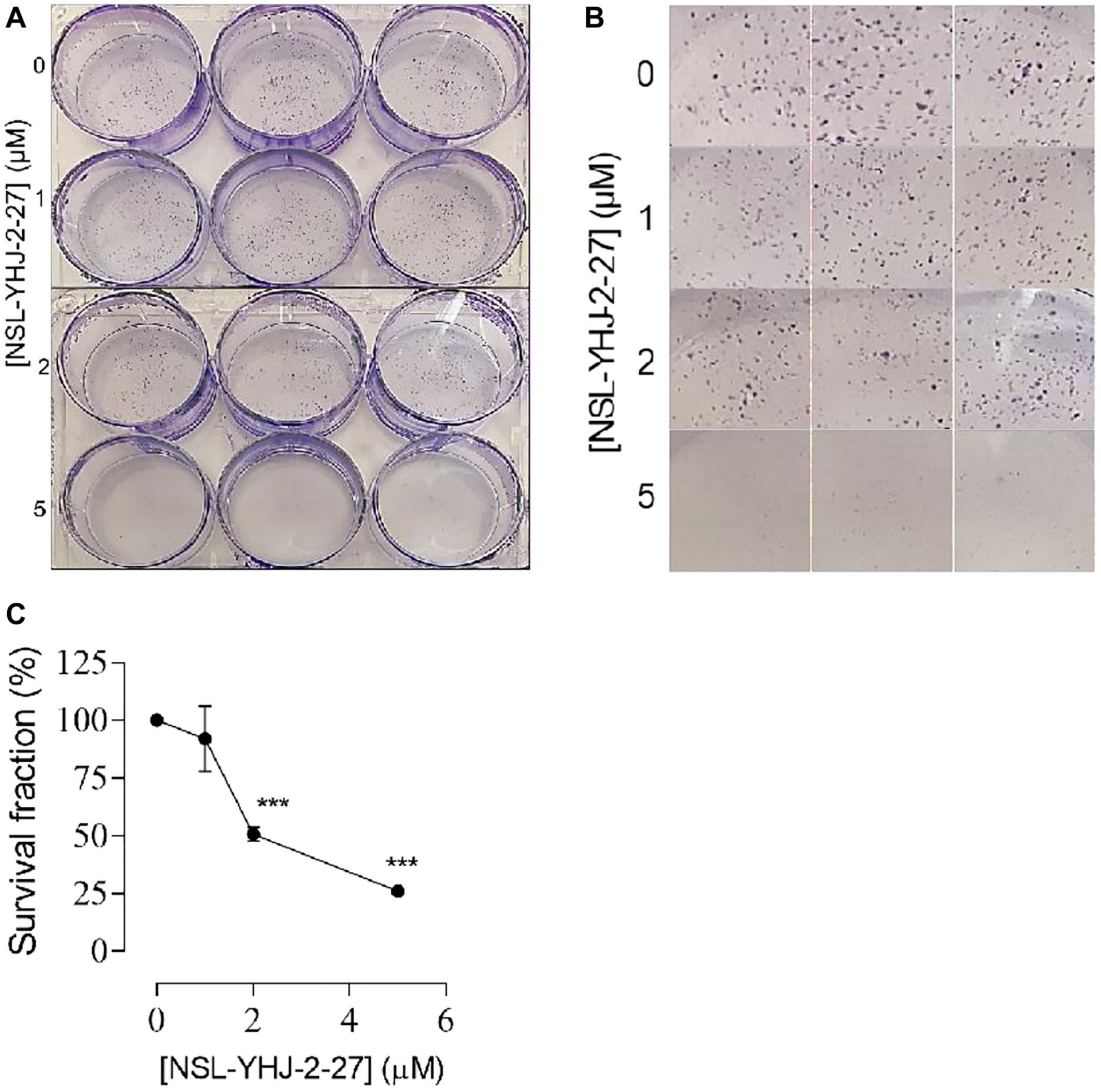
NSL-YHJ-2-27 inhibits LTLT-Ca colony formation. (**A**, **B**) Cells were initially grown in 25 cm^2^ flasks and treated with the indicated concentrations of PCAIs. Then, 1000 cells from the respective flasks were seeded onto a 6-well plate and left to grow for 12 days. These were then fixed and stained as described in the methods section. (**C**) Colonies with more than 20 cells were counted and the survival fractions were calculated and plotted. Significance was calculated from three independent experiments. Statistical significance (^***^ *p* < 0.001) was determined by one-way ANOVA with post hoc Dunnett’s test.

Rapid proliferation of cancer cells leads to the formation of colonies resulting in tumors. Our results show that NSL-YHJ-2-27 significantly inhibited the formation of colonies as indicated by the cell survival fractions. The number of colonies observed relative to the density of cells seeded at 1,000 cells/well decreased by 49% and 74% in wells initially treated for 48 h with 2 and 5 μM of PCAIs, respectively, before further incubation without treatment for 12 days in complete growth medium (Figure 8). No significant effect was observed for cells treated with 1 μM NSL-YHJ-2-27. These results indicate that at suitable concentrations, the effect of treatment is persistent even after treatment is withdrawn, underlining the irreversibility of the effects at the effective concentrations.

### NSL-YHJ-2-27 impedes the migration and invasion of LTLT-Ca cells and spheroids

**Figure 9:**
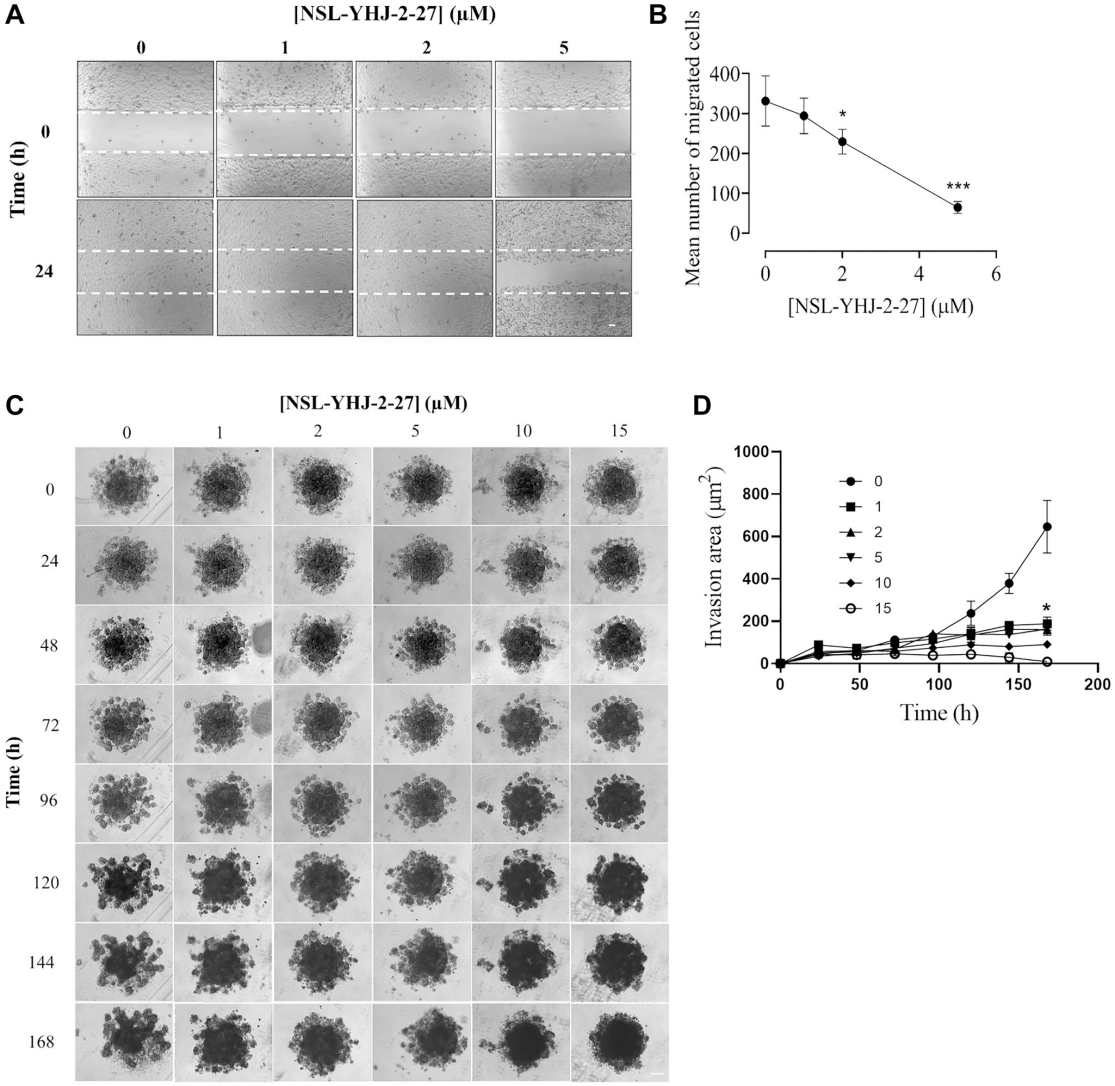
NSL-YHJ-2-27 inhibits LTLT-Ca cells migration and LTLT 3D spheroids. (**A**) The ‘wound’ was formed using cell culture inserts (ibidi) and were grown to confluency in a 24-well plate. These were then treated with the indicated concentrations of PCAIs. Cell culture and treatments were done in triplicates and closure of the wound was monitored for 24 h, capturing images at 0 and 24 h using the Nikon Ti Eclipse microscope at 4x magnification (scale bar = 100 μm). (**B**) The cells that migrated into the wound were counted and plotted against concentration. Statistical significance (^*^ *p* < 0.05, ^***^ *p* < 0.001) was determined by one-way ANOVA with post hoc Dunnett’s test (**C**) LTLT-Ca preformed spheroids in experimental media were treated with the (0, 1, 2, 5, 10 and 15 μM) concentrations of NSL-YHJ-2-27. PCAIs-treated Matrigel was then added to the spheroids and allowed to solidify for 30 minutes. Images were then taken at 0 h and every 24 h for seven days, using a Nikon Ti Eclipse microscope at 4X magnification. Time-dependent changes in spheroid invasion regions were measured for each treatment concentration and quantified using NIS-Elements AR version 4.30. (**D**) The area invaded by the spheroids at each concentration was measured and plotted against time points. Invasion area against concentration of PCAIs at each time point was analyzed with Two-Way ANOVA with Dunnett’s posthoc test, statistical significance (^*^ *p* < 0.05).

Migration of cells is a natural process but for cancer cells it implies progression of the cancer. Previous studies using PCAIs demonstrated profound inhibition of cell migration on the different cell lines tested [35–37, 43, 45]. Mitigating this cancer hallmark is essential for stopping the cancer cells from spreading to form secondary metastatic lesions. Polyisoprenylated proteins such as RHOA, CDC42 and RAC1 are involved in the actin filaments remodeling necessary for cell migration. Molecules such as the PCAIs that are designed to disrupt the G-proteins’ interactions may disrupt the cytoskeletal structures and thus the cell migration and invasion. The results here show that the PCAIs significantly inhibited the migration of the LTLT-Ca cells by 31% and 80% after 24 h treatment with 2 and 5 μM PCAIs, respectively (Figure 9A, 9B). Also, LTLT-Ca cells were grown into 3D spheroids to more closely emulate tumors. These were then incubated with or without the PCAIs to determine their effect on LTLT cell invasion. After 168 h of incubation, mini spheroids signifying invasion into Matrigel were observed in the periphery of the original untreated control spheroids (Figure 9C). Spheroids treated with NSL-YHJ-2-27 showed significant 86 and 96% inhibition of invasion at 10 and 15 μM, respectively after 168 h exposure (Figure 9D).

### NSL-YHJ-2-27 disrupts actin filaments and depletes vinculin levels

**Figure 10:**
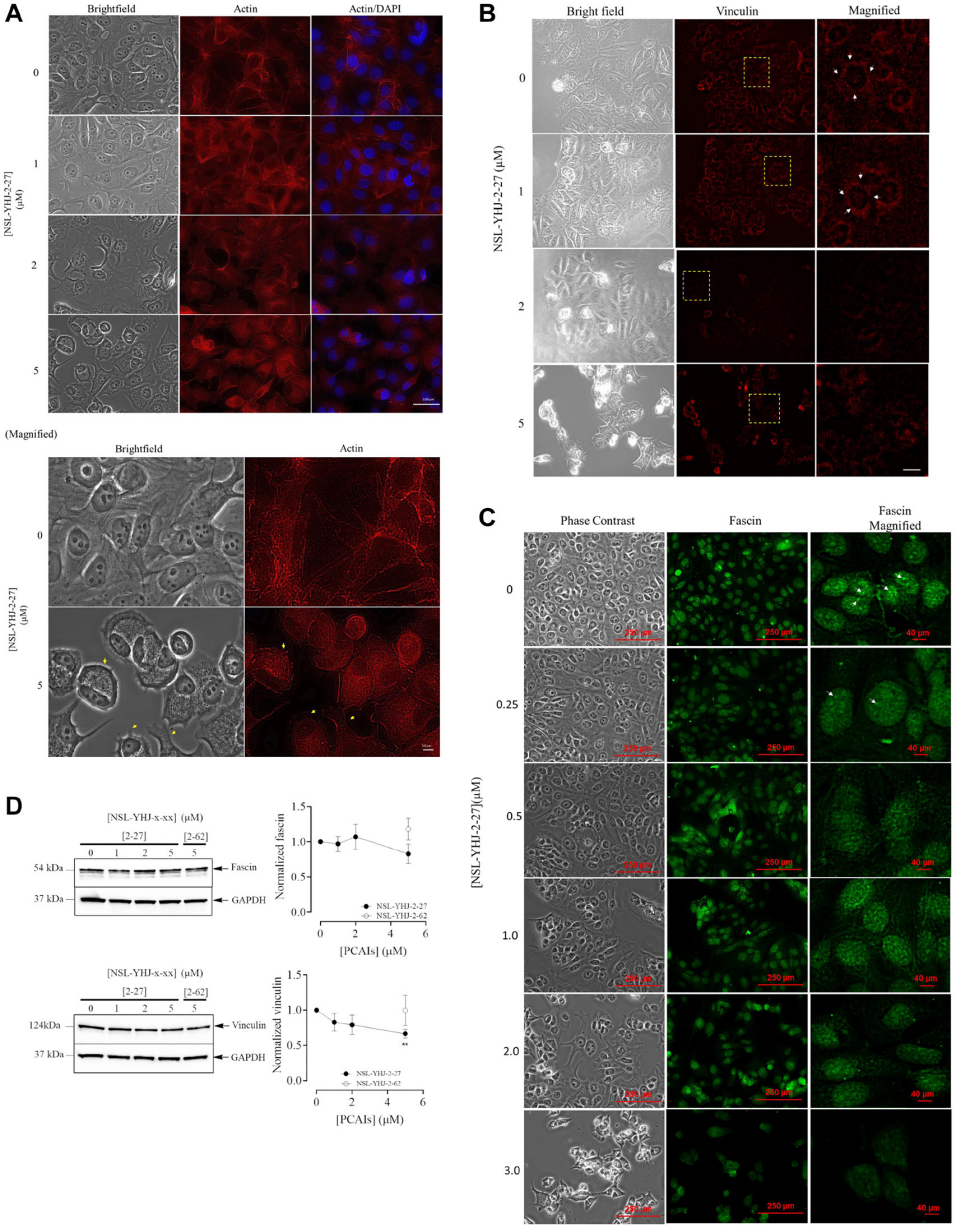
PCAIs treatment collapses actin filaments and delocalizes focal adhesion proteins. Cells were grown in 8-well ibidi μ-slides overnight and treated with the respective concentrations of PCAIs for 48 h, then fixed and permeabilized. Images of the cells treated with 0 and 5 μM NSL-YHJ-2-27 are shown at higher magnification whereby yellow arrows indicating retracting lamellipodia indicative of collapsing actin filaments. (**A**) Triplicates of the fixed cells were stained with Alexa Fluor^™^ 568 Phalloidin for actin filaments. Yellow arrows indicate retracting lamellipodia indicative of the collapsing actin filaments (magnified images). (**B**) Vinculin punctates were probed using vinculin antibody and visualized using rabbit IgG Alexa Fluor 555 conjugate. White arrows indicate vinculin punctates. (**C**) Fascin was probed with fascin antibody and visualized using mouse IgG Alexa Fluor 488. White arrows indicate defined fascin spots. Images were captured using Keyence BX-X800 microscope at 40X magnification. (**D**) Cells treated for 48 h with the indicated concentrations of NSL-YHJ-2-27 or 5 μM of the non-farnesylated PCAIs analog, NSL-YHJ-2-62. Lysis of the cells and analysis by western blotting for vinculin and fascin were conducted as described in the methods. Western blot images and plots of chemiluminescence intensities of the bands following quantification using Image Lab Software normalized against GAPDH against concentration. Data are representative of three independent experiments. Statistical significance (^**^ *p* < 0.01 in relation to the negative control, 0 μM) was determined by one-way ANOVA with post hoc Dunnett’s test.

Prominent features of cell migration and invasion are the formation and remodeling of the actin cytoskeleton [44]. Serving as the structural framework for the migrating cells, F-actin works with such focal adhesion proteins as fascin and vinculin [46]. The PCAIs disrupted the expansive network of actin filaments in cells treated with 5 μM of NSL-YHJ-2-27. As seen in Figure 10A, the actin filaments retracted, resulting in changes in cell morphological features. Vinculin punctates serve as the anchoring points for actin filaments during cell movement. Concurrent with actin staining, the vinculin punctates were prominently observed in untreated cells and those treated with 0 and 1 μM of NSL-YHJ-2-27 but noticeably depleted and less defined in those treated with the higher 2 μM and 5 μM concentrations (white arrows in Figure 10B). Defined and pronounced fascin spots are observed at 0, 0.25 and 0.5 μM of NSL-YHJ-2-27 but become less pronounced and “smudgy” at higher concentrations of 1, 2 and 3 μM (white arrows in Figure 10C). Moreover, western blot results revealed a significant 33% decrease in vinculin levels (Figure 10D).

## DISCUSSION

The role of estrogen in the occurrence, malignant progression, and prognosis of breast cancer has long been recognized [47]. Epidemiological studies have demonstrated that the mitogenic and tumorigenic role of estrogens is pronounced in women with prolonged exposure to the hormone, while it is diminished in women with non-functional ovaries [48]. Approximately 70% of breast cancers are hormone receptor positive (HR^+^) which are commonly characterized by their dependence on ER signaling [49]. In fact, a decline in the mortality rate of the majority of ER^+^ breast cancer cases in recent years have been due to the success of endocrine therapies with aromatase inhibitors such as letrozole, anastrozole, and exemestane and anti-estrogens such as tamoxifen. Although these are some of the most effective treatment options for ER^+^ breast cancers [5], resistance occurs in about 30% of cases [50]. Although previous reports on LTLT-Ca cells have demonstrated several mechanisms responsible for the transition from letrozole sensitivity to letrozole resistance including activation of the MAPK signaling pathway [9, 13], increased cancer stem cell formation [51], induction of EMT [52], estrogen independence and increased cell motility [10], effective strategies to combat resistance to endocrine therapies remains a significant clinical challenge. While several advances have been made including the use of the cdk4/6 inhibitors, not all patients respond to this therapy. Identification of the resistance mechanisms thus opens avenues for the development of novel therapies that specifically target cancer progression drivers in the resistant phenotypes. MAPK pathway involvement in letrozole resistance opens an avenue for the investigation of anti-RAS agents such as the PCAIs since several studies have implicated RAS activation in the reduction of estrogen dependence and invasiveness in breast cancer [53–55]. A more recent investigation on drug resistance in hormone-dependent MCF-7 cells revealed that transfection of MCF-7 cells with activated *RAS* oncogene (*H-Ras*^G12V^) resulted in the survival of the cells in hormone-depleted culture medium. The transfected MCF-7 cells demonstrated increased resistance to paclitaxel, doxorubicin, and 5-fluorouracil, which are commonly used chemotherapeutic agents for treating breast cancer patients. Furthermore, between the two major RAS downstream pathways, PI3K/Akt and MEK/MAPK, it was determined that PI3K/AKT pathway is more involved in the RAS activation-induced drug resistance of the transfected MCF-7 cells [27]. Since RAS is at the core of major intracellular signaling pathways involved in cell growth and motility, it tends to play a default role in many oncogenic phenomena. The design of the PCAIs to mimic the posttranslational modifications of RAS and other G-proteins is based on the premise that the PCAIs compete for the polyisoprenyl binding sites of RAS and related proteins, thereby disrupting their interactions while exposing them to degradation or secretion from the cells [43] and most importantly blocking their functions. The potency of the PCAIs against various cancer cell lines has previously been reviewed [33]. Although lung cancer cell lines demonstrate a decrease in KRAS levels, activation of the MAPK pathway kinases MEK1/2, ERK 1/2, and p90RSK is observed after treatment with the PCAIs [39, 43]. Here, exposure of LTLT-Ca cells to PCAIs also stimulates the phosphorylation of MEK1/2, ERK 1/2, and p90RSK. We believe that the observed apoptosis in the cells is due to the perturbation of the MAPK signaling kinases, since prolonged stimulation of ERK 1/2 along with p90RSK have been associated with programmed cell death, senescence, and cell cycle arrest [56–59]. This observation is further substantiated by the increase in active caspase 3/7 levels. Furthermore, the significant activation of AKT phosphorylation makes it even more challenging to pinpoint the molecular changes that actually induce the death of the cells. While AKT has been reported as an anti-apoptotic protein [60], AKT activation has been shown to induce apoptosis [61]. Other studies linking AKT activation with cell death is attributed to the increased oxygen consumption and production of ROS that damage DNA, proteins and lipids [42]. Rather than protect cells from apoptosis, AKT sensitizes them to ROS-induced apoptotic cell death [42]. Use of the CaspaTag reagent, a specific fluorescently labeled irreversible caspase 3/7 inhibitor, demonstrates the increase of active caspase 3/7 and apoptosis in the PCAIs-treated cells. AKT phosphorylation also suppresses the expression of genes that degrade ROS and promote cell survival (reviewed in [41]). In other breast cancer cell lines, the PCAIs similarly activate MEK/MAPK and PI3K/AKT pathways [40]. While it is not clear which of the two activated pathways is causing the apoptosis, experiments with specific inhibitors of enzymes of the two pathways will ultimately contribute to answering these questions. The findings are however consistent with our expectations for the PCAIs to alter signaling by KRAS and similarly post-translationally modified G-proteins such as RAC1, RHOA and CDC42. Why the PCAIs activate these pathways rather than suppress them remains the subject of investigation. One possibility is that the PCAIs may functionally replenish KRAS and other G-protein functions by binding to effectors that rely on the polyisoprenylated cysteine for interaction. It is also possible that the PCAIs may displace the G-proteins from their molecular chaperones rendering them free for binding interactions that activate downstream signaling intermediates of the MAPK and PI3K/AKT pathways.

When breast cancer cells become resistant to letrozole treatment, they commonly develop into more malignant forms due to the enhanced growth factor signaling and increased cell motility and invasiveness [52]. Thus, the PCAIs’ significant negative impact on LTLT-Ca cells proliferation and colony formation implies that the PCAIs may have a significant therapeutic benefit in addressing the letrozole resistance problem. Because resistance also leads to the transition of the cells from an epithelial to a mesenchymal phenotype making them more migratory [62], it is essential that the anticancer agent be able to disrupt the cells’ machinery for migration. The actin filaments are directly involved in the cytoskeletal functions of the cells, such as movement and structure, regulated by the RAS-related small GTPases of the RHO family that includes RAC, RHO, and CDC42 [63–65]. Aberrant activation of these proteins is involved in cancer progression [65–67]. For instance, elevated RAC1 expression is associated with aggressiveness and poor prognosis in colorectal, breast, lung, thyroid, and pancreatic cancers [68–72]. We have shown previously that the PCAIs suppress the levels of RAC1, RHOA, and CDC42 in cancer cell lines [43]. A prominent characteristic change observed in the LTLT-Ca cells after exposure to PCAIs is the cell shrinkage, retraction from the periphery resulting in rounding and loss of adherent properties. In agreement with the phalloidin staining, F-actin filaments in the treated LTLT-Ca cells tend to retract and shrink. Furthermore, cell motility also requires focal adhesion (FA) proteins, which serve as anchors or contact points for the cells with the extracellular matrix [73]. Here, fascin and vinculin levels decreased after PCAIs treatment. The disruption of the actin filaments and disappearance of vinculin punctates in cells treated with 5 μM NSL-YHJ-2-27 further explain the observed PCAIs inhibition of cell migration and possibly the disaggregation of compact spheroids making the spheroids appear more voluminous at higher concentrations. These results agree with our previous findings on non-small cell lung, pancreatic, and triple-negative breast cancer cells, suggesting the involvement of PCAIs at inducing cell rounding and suppressing cell movement [35, 37, 38, 43]. These effects on focal adhesion, cell shape, anchorage and migration also suggest anoikis as a contributing mechanism of PCAIs-induced cell death as anoikis has been reported to be influenced by the PI3K/AKT signaling pathway [74, 75].

Resistance to therapy hinders the full potential of chemotherapeutic agents. It is thus vital to understand the resistance mechanisms in order to counteract them. Developing targeted therapies that specifically impact associated resistance drivers such as oncogenic RAS and hyperactive G-proteins is therefore imperative. The PCAIs constitute a novel group of anticancer agents designed to treat cancers driven primarily by KRAS and related proteins or those that rely on them as resistance genotypes, such as the drug-resistant forms of breast cancer that are the subject of the present study. This study shows the potential applicability of the PCAIs as effective therapies to AI- resistant breast cancer therapy.

## MATERIALS AND METHODS

### Materials

LTLT-Ca cells [9] were kindly provided by Dr. Syreeta L. Tilghman. Primary antibodies specific to proteins: total MEK 1/2 (Cat #8727S), phosphorylated MEK 1/2 (p-MEK 1/2, Cat #9154), total ERK 1/2 (Cat#4695S), phosphorylated ERK 1/2 (p-ERK1/2, Cat #4370), total P90RSK (Cat #9355S), total AKT (Cat #4691S), phosphorylated AKT(pAKT(Ser473)(Cat#4060S), phosphorylated P90RSK (p-P90RSK, Cat #11989), RAC1/2/3 (Cat #2465), CDC42 (Cat #2462), vinculin (Cat #18799), fascin (Cat #54545), caspase 7 (Cat #9492S), HER2/ErbB2 (Cat #60388S), BAX (Cat #5023S), P-p53 (9284S), PARP (9542T), p-ERα (2511T), t-ERα (13258S); secondary antibodies: anti-mouse IgG, HRP-linked Antibody (Cat #7076), anti-rabbit IgG, HRP-linked Antibody (Cat #7074) and housekeeping protein, GAPDH (Cat #5174) were purchased from Cell Signaling Technology (Danvers, MA, USA). CaspaTag^™^ Caspase-3,7 *In Situ* Assay Kit (Cat # APT403) was purchased from Sigma Aldrich (Burlington, MA, USA). The PCAIs used in this study (Table 1) were synthesized in our lab as previously described [43].

**Table 1:** Structures of the PCAIs analogs used in the study

Compound	Structure
NSL-YHJ-2-27	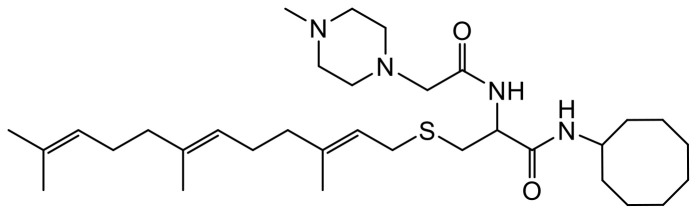
NSL-YHJ-2-40	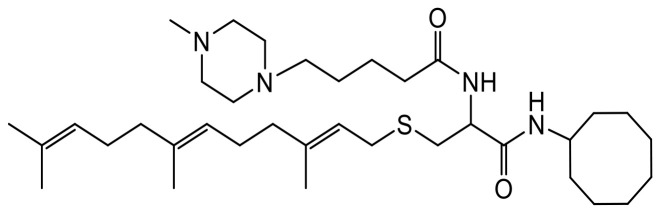
NSL-YHJ-2-44	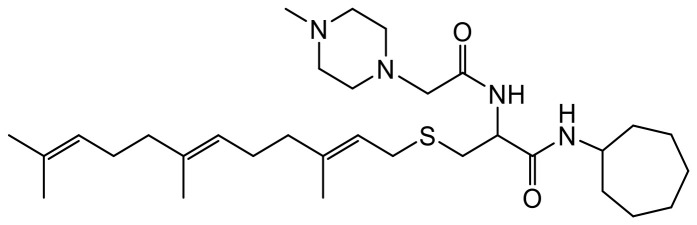
NSL-YHJ-2-62	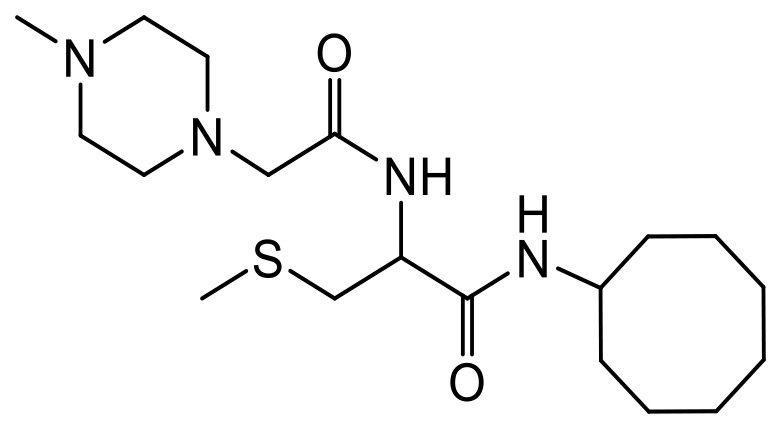

### Cell culture

LTLT-Ca cells were cultured in MEM (Richter’s modification) (Cat # A1048801 Life Technologies, Carlsbad, CA, USA). The media were supplemented with 10% charcoal-stripped heat-inactivated fetal bovine serum (CS-FBS), 100 U/mL penicillin and 100 μg/mL streptomycin, 750 μg/mL Geneticin^™^ Selective Antibiotic (G418 sulfate) (Cat # 10131027) (Life Technologies, Carlsbad, CA) 1 μM letrozole (Cat # L6545-50MG) (Sigma-Aldrich, St. Louis, MO, USA). The cells were cultured at 37ºC in 5% CO_2_/95% humidified air. In all experiments, the cells were grown in experimental medium which is the base medium supplemented with 5% CS-FBS.

### Cell viability assay

Four different PCAIs analogs (NSL-YHJ-2-62, NSL-YHJ-2-27, NSL-YHJ-2-40, and NSL-YHJ-2-44) were investigated for their effects on cancer cell viability. Briefly, LTLT-Ca cells were plated into 96-well culture plates (Genesee Scientific, San Diego, CA, USA) at a density of 1 × 10^4^ cells/well in experimental medium. Adherent cells were treated with varying concentrations of PCAIs (0.5, 1, 2, 5, 10, 20, 50 μM) in quadruplicates for each concentration. Acetone (carrier solvent, 1%), the vehicle solvent, was used for the control (0 μM) treatment. After 24 h of the first treatment, treatments were repeated with the respective concentrations of the compounds. After 48 of exposure to the compounds, the resazurin reduction assay was performed by adding 0.02% of resazurin reagent to each well. The plates were incubated at 37ºC in 5% CO_2_/95% humidified air for 2 h. The fluorescence intensities were determined using SoftMax Pro Reader version 5.4 for Windows (Molecular Devices, San Jose, CA, USA), where the excitation frequency was set at 544 nm and emission at 590 nm. The percentages of the fluorescence in the treated cells relative to the controls (0 μM) were plotted against the logs of treatment concentrations in a non-linear regression curve fit using GraphPad Prism version 8.0 for Windows (San Diego, CA, USA) to determine the EC_50_ values for each compound.

### Effect of PCAIs on proliferation and morphology of LTLT-Ca cells

Cell proliferation assays were conducted to determine the impact of PCAIs on the proliferation of LTLT-Ca cells. The LTLT-Ca cells were seeded at a density of 2 × 10^5^ cells/well in a 6-well plate and incubated overnight to adhere. The baseline number of cells was measured. PCAIs treatment was conducted twice, once for the 24-h treatment and another for the 48-h exposure with 0, 1, 2, and 5 μM. Viable cells were counted after PCAIs treatment using the Countess II cell counter (Life Technology Corporation, Grand Island, NY, USA). The data were calculated and plotted using GraphPad prism version 8.0 (San Diego, CA, USA) to determine the effects on proliferation. Morphological alterations in the cells post-PCAIs treatments were observed, and images were obtained using Keyence BZ-X800 microscope (Keyence Corporation of America, Itasca, IL, USA) at 20× magnification.

### Effect of PCAIs on RAC1 and CDC42 levels and the phosphorylation of Mitogen-Activated Protein Kinase (MAPK) and PI3K/AKT pathway kinases

To evaluate the PCAIs’ effect on the phosphorylation of MAPK and PI3K/AKT enzymes and the levels of RAC1 and CDC42, western blot analysis was performed. Cells were prepared, treated with PCAIs as described in preceding section, and the cells lysed 24 h post treatment. The experimental media were removed from the dishes, and the cells washed thrice with 1× PBS to remove remnants of dead cells in the media. The cells were lysed by incubating them in RIPA lysis buffer supplemented with 0.1% v/v protease/phosphatase inhibitors cocktail (Cell Signaling Technology, Danvers, MA, USA). Protein concentrations in the lysates were determined using Quick Start™ Bradford protein assay (Bio-Rad, Hercules, CA, USA).

### Western blot analysis

The samples were prepared by mixing 30 μg of the lysates with XT sample buffer and XT reducing agent (Bio-Rad, Hercules, CA, USA) followed by boiling for 5 min. The reduced proteins were separated by SDS-PAGE on 4–12% Criterion^™^ XT Bis-Tris protein gels. The proteins were then transferred onto Trans-Blot Turbo midi 0.2 μm nitrocellulose membranes (Bio-Rad, Hercules, CA, USA). Membranes were incubated in OneBlock^™^ western-CL blocking buffer (Genesee Scientific, San Diego, CA, USA) for 1 h at room temperature. The membranes were then treated overnight at 4^o^C with the respective primary antibodies. The membranes were the washed thrice using 1× TBST (Genesee Scientific, San Diego, CA, USA). The membranes were then treated with either HRP-linked anti-rabbit or anti-mouse IgG at room temperature for 1 h. Immunoreactive bands were visualized by immersing the membranes in the chemiluminescent substrate, Radiance Plus (Azure Biosystems, Dublin, CA, USA) imaging with ChemiDoc XRS+ System (Bio-Rad, Hercules CA, USA). The chemiluminescent intensities of the target proteins were quantified using Image Lab 6.0 (Bio-Rad, Hercules CA, USA). All quantified proteins were normalized against GAPDH and the respective total proteins for the determination of protein phosphorylation levels as previously described [76]. Three independent experiments were performed, and the results plotted against treatment concentrations using GraphPad Prism version 8.0 for Windows (San Diego, CA, USA).

### Effect of PCAIs on induction of reactive oxygen species (ROS)

LTLT cells were seeded with 1 × 10^5^ cells/per well in 500 μL experimental media in a 24 well plate. Cells were treated in triplicates with NSL-YHJ-2-27 (0, 0.5, 1, 3 and 5 μM) for 48 h. The drug containing media was removed and cells were washed once with serum free media. After that, 10 mM stock solution 2,7 dichlorofluorescein diacetate (DCFH-DA) (St. Louis, MO) was diluted to 25 μM working solution using prewarmed experimental media. Approximately 500 μL of DCFH-DA working solution was pipetted into each well and incubated for 40 min. Then the DCFH-DA working solution was removed, cells washed twice with experimental media and finally were washed again once with 1X PBS. Representative fluorescent images were taken for each concentration at 10X magnification using the Keyence BZ-X800 series microscope. The mean fluorescent intensities per cell for each concentration, indicative of ROS generation by PCAIs treatment, was measured, analyzed with the Keyence BZ-X800 analyzer and data plotted with GraphPad Prism. One-Way ANOVA was used to determine the statistical significance between the controls and the different PCAIs treatment concentrations.

### Effect of PCAIs on LTLT-Ca spheroids

Spheroid LTLT-Ca cells were grown to determine if NSL-YHJ-2-27 induces apoptosis in 3D-cultured cells. The spheroids were prepared by seeding 5 × 10^3^ cells/well into 96U round bottom Nunclon Sphera Plates. The cells were grown for 48 h to obtain compact spheroids. PCAIs (0, 5, 10, 20 and 50 μM) treatments were conducted for up to 144 h while bright field images were taken every 24 h using Nikon Ti Eclipse microscope (Nikon Instruments Inc., Melville, NY, USA) at 4× magnification. In another experiment, the spheroids were treated for 48 h followed by staining with 5 g/mL of acridine orange/ethidium bromide (AO/EB) solution to distinguish live from dead cells. Fluorescent intensity ratios of AO/EB for the spheroids were determined and plotted against PCAIs concentrations using GraphPad Prism version 8.0 (San Diego, CA, USA). Three replicates were prepared for each treatment concentration.

### Fluorescence microscopy analysis on PCAIs-induced caspase activity

Active caspases such as caspases 3 and 7 are essential for cells to undergo apoptosis. Using the CaspaTagTM caspase-3/7 *in situ* fluorescent kit, the PCAI’s ability to induce active caspase activity was assessed. LTLT-Ca (1 × 10^5^ cells/mL) were seeded onto an 8-well μ slide plate (ibidi) and allowed to adhere overnight, as per the manufacturer’s instructions. After a 24 h incubation, the media were removed and replaced with treatment media containing 0 μM, 0.25 μM, 0.5 μM, 1 μM, 2 μM, 3 μM, and 5 μM of NSL-YHJ-2-27. The treatment was repeated after a 24 h incubation period, followed by another 24 h incubation. The CaspaTag FLICA reagent (1:30 dilution) was added to the experimental media in place of the treatment media. The 8-well μ slide plates from Ibidi were incubated in 5% CO_2_ for 1 h at 37^o^C. After that, the cells and media were removed using 2 mL of 1X wash buffer. The Keyence BZ-X800 microscope was used to examine the cells. GraphPad Prism 8.4.3 was used to create a graph after images were taken and the BZ-800 analyzer was used to quantify the mean fluorescence intensities per cell. Western blotting was also used to analyze treated cells to determine the levels of apoptotic markers.

### Inhibitory effect of PCAIs on LTLT-Ca colony formation

To determine the effect of the PCAIs on colony formation, 5 × 10^5^ cells/well were seeded into a T-25 cm^2^ flasks. The cells were treated with 0, 1, 2, and 5 μM of PCAIs. After 48 h treatment, the cells were trypsinized and seeded at a density of 1,000 cells/well in 6-well plates and maintained for 12 days in complete growth medium without any further treatments. The cells were then fixed in a solution of 1:7 acetic acid in methanol followed by staining crystal violet (1% solution in methanol). The colonies were observed under a microscope and those with 20 or more cells were manually counted. The equations used to calculate the surviving fraction (SF) is as follows: Plating efficiency (PE) = number of colonies formed/number of cells plated × 100 (for both treated and control cells) and SF = PE of treated cells/ PE of untreated cells × 100.

### Effect of PCAIs on LTLT-Ca cell migration and 3D spheroid invasion

The PCAIs’ effect on the migration of LTLT-Ca cells was evaluated using the wound healing technique. Cell-free zones (wounds) were created using ibidi culture inserts (Cat # 80209, ibidi USA, Inc. Fitchburg, WI, USA) in 12-well plates to produce two confluent monolayers of cells separated by the ‘wound’. Cells in serum-free media were seeded at a density of 3 × 10^4^ cells/well into the wells and allowed to adhere overnight. The inserts were then removed, and the serum-free media were replaced with fresh experimental media after which the cells were treated with varying concentrations of the PCAIs (0, 1, 2, and 5 μM). Closure of the ‘wound’ was monitored by taking images at 0 and at 24 h images using the Nikon Ti Eclipse microscope at 10× magnification. The cells that migrated into the wound were counted using the Nikon NIS-Elements software version 4.30.02 (Nikon Instruments Inc. Melville, NY, USA). Migrated cells were counted from three distinct squares of equal area of the ‘wound’ for each replication. Three replicates for each concentration were prepared. Data were analyzed using GraphPad Prism version 8.0 for Windows (San Diego, CA, USA). To create spheroids, LTLT-Ca monolayer cells were suspended in complete medium, seeded in 96U Nunclon Sphera plates (Thermo Scientific, Waltham, MA) at a density of 1.0 × 10^4^ cells/mL using 200 μL growth media, and then incubated for 72 h at 37°C/5% CO_2_. After 72 h, half of the medium was removed, and the remaining was treated with acetone (vehicle) or NSL-YHJ-2-27 (0-10 μM) dissolved in acetone. Matrigel (Corning, NY, 100 μL) mixed with acetone (vehicle, 2 μL) or 1 to 10 μM PCAIs contained in 1 μL of acetone were put into each well and incubated at 37°C/5% CO_2_ for 30 minutes for solidification. Images were captured every 24 hours for 7 days with the Nikon Eclipse microscope. Time-dependent changes in spheroid invasion areas were measured for the control and treated spheroids using NIS- Elements AR version 4.30. The invasion areas for spheroids of LTLT-Ca cancer cells were determined for each treatment concentration, (invasion area at each time point - invasion area at 0 h for each concentration. Graphs illustrating invasion areas against each time point were plotted using GraphPad Prism version 8 on windows.

### Effect of PCAIs on F-actin filaments, fascin and vinculin punctates

To evaluate the effect of the PCAIs on the formation and organization of actin fibers, actin staining was conducted using Alexa Fluor^™^ 568 Phalloidin (Thermo Fisher Scientific, Waltham, MA, USA). LTLT-Ca cells suspensions containing 10,000 cells were seeded into each well of μ-slide 8-well glass bottom (ibidi USA, Inc. Fitchburg, WI, USA) and incubated overnight at 37ºC in 5% CO_2_/95% humidified air. When the cells completely adhered to the wells, they were treated with either the vehicle solvent, acetone (0 μM) or NSL-YHJ-2-27 (5 μM) for 48 h. They were then fixed in 4% paraformaldehyde for 10 min, permeabilized in 0.5% Triton X-100 for 10 min, stained with phalloidin 568 and Hoechst for 30 min at room temperature in the dark. Because actin works in conjunction with vinculin and fascin during cell movement, the PCAIs effect on vinculin punctates and fascin was determined by immunocytochemistry. Cells were grown in μ-slide 8-well glass bottom and treated with respective concentrations of NSL-YHJ-2-27 (0, 1, 2, and 5 μM). The cells were then fixed in 4% paraformaldehyde for 10 min, permeabilized in 0.5% Triton X-100 for 10 min. Blocking was done for 1 h using 1% BSA in room temperature. To probe for fascin and vinculin punctates, the fixed cells were incubated in 1% BSA containing antibody against fascin and vinculin, respectively, overnight in the cold room. Rabbit IgG Alexa Fluor 555 (Thermo Fisher Scientific, Waltham, MA, USA) was used to label vinculin while mouse IgG Alexa Fluor 488 (Thermo Fisher Scientific, Waltham, MA, USA) was used for fascin. Fluorescent images were captured with Keyence BX-X800 microscope (Keyence Corporation of America, Itasca, IL, USA) at 40× magnification. Western blot assay was also performed to determine vinculin and fascin levels after PCAIs treatment. Preparation of cells and lysates were conducted as described in Section 2.6.

### Statistical analysis

To determine statistical significance, the values of each treatment group were compared to the respective controls either by One-Way ANOVA with Dunnett’s post-hoc test or Two-Way ANOVA with Dunnett’s multiple comparisons test using GraphPad Prism version 8.0 for Windows (San Diego, CA, USA) and ^*^ *p* < 0.05; ^**^ *p* < 0.01; ^***^ *p* < 0.001; ^****^ *p* < 0.0001 were considered significant as shown in the figure legends.
